# Effects of Cocoa Butter and Cocoa Butter Equivalent in a Chocolate Confectionery on Human Blood Triglycerides, Glucose and Insulin

**DOI:** 10.3390/foods9040455

**Published:** 2020-04-08

**Authors:** Rina Yu Chin Quek, Elaine Wan Yi Peh, Christiani Jeyakumar Henry

**Affiliations:** 1 Clinical Nutrition Research Centre (CNRC), Singapore Institute for Clinical Sciences (SICS), Agency for Science, Technology and Research (A*STAR) and National University Health System, 14 Medical Drive, Singapore 117599, Singaporeelaine_peh@sifbi.a-star.edu.sg (E.W.Y.P.); 2 Department of Biochemistry, Yong Yoo Lin School of Medicine, National University of Singapore, 8 Medical Drive, Singapore 117596, Singapore

**Keywords:** cocoa butter, shorea robusta (sal seed oil), triglycerides, glucose, insulin, oleogel

## Abstract

Given the rising trend in the consumption of chocolate confectioneries, the shortage in cocoa butter (CB) production remains a constant threat to food manufacturers. Therefore, exploring alternative plant sources of CB is essential. Sal fat, obtained from seed kernels of trees, has the potential to substitute CB in chocolate confectioneries. The primary aims of this randomised controlled, crossover trial was to compare the glycaemic, insulinaemic and lipidaemic response of two different oil types (CB and Sal fat) in people and the effects of these oils in two physical forms (liquid and oleogel). Seventeen healthy male participants (age 24.73 ± 2.63, height 173.81 ± 7.24 cm, weight 65.85 ± 8.06 kg, BMI 21.73 ± 1.65 kg/m^2^) completed the study. There were no significant differences in blood glucose iAUC (*p* = 0.995), plasma insulin (*p* = 0.760) and triglyceride (TG) (*p* = 0.129), regardless of oil type consumed. When comparing incremental area under the curve (iAUC) of insulin and TG between the different forms (liquid or oleogel), oleogel was found to be significantly lower (*p* = 0.014 and *p* = 0.024 respectively). Different types of oil transformed into oleogels are effective in reducing postprandial insulinaemia and lipidaemia. Sal fat, although not metabolically different from CB, can be an acceptable substitute for CB in the production of chocolate confectioneries.

## 1. Introduction

For several decades now, cocoa beans (*Theobroma* cacao) have been reported to be a natural source of antioxidant and contain flavonoids such as flavan-3-ols, procyanidins and epicatechin equivalents, which have been reported to be beneficial for cardiovascular health [1]. Cocoa butter (CB), a triglyceride (TG) found naturally in cocoa beans, is a key ingredient in the manufacturing of chocolate confectioneries. It is responsible for the melting properties of chocolate, consisting predominantly of fatty acids such as palmitic acid (C16:0), stearic acid (C18:0), oleic acid (C18:1) and linoleic acid (C18:2), with low levels of lauric acid (C12:0) and myristic acid (C14:0) [2]. Fatty acid profiles are categorized into saturated, monounsaturated and polyunsaturated fats. Palmitic, stearic, lauric and myristic acid are considered saturated fatty acids, oleic acid is a monounsaturated fatty acid while linoleic acid is a polyunsaturated fatty acid. Cocoa, although manually processed in the initial stages, is subsequently processed mechanically. The cocoa beans are first made into cocoa liquor and then into CB or powder for producing chocolates and other products [3].

Due to cocoa’s unique chemical and physical properties, CB is highly valued and costlier than other vegetable fats and oil. The 2019 Global Market report indicated that the demand for CB is expected to grow at a compound annual growth rate of 7.3% in the next five years, yielding 16.32 billion USD (3). Market researchers have predicted that given the rising trend in the consumption of chocolate confectioneries, the poor yield returns and the low wages of working in a cocoa farm, the shortage in CB production will remain a constant threat to food manufacturers [3,4]. Therefore, these researchers have spurred the food manufacturing industry to look towards producing cocoa butter equivalents (CBEs), an alternative from plant sources, to either partially or completely replace CB in food products for both economic and technological reasons [5].

CBEs are used in food products such as chocolates, ice cream and bakery products such as cakes, biscuits and breads [6]. Some examples of CBEs include Sal fat, mango seed fat, shea butter and palm oil that have undergone various methods of processing such as chemical or enzymatic interesterification or fractionation and blending [7,8,9].

Sal fat (*Shorea robusta)* is obtained from seed kernels of trees grown in places like Borneo, Java, Malaysia, India and the Philippines. Historically, it was primarily used as a cooking oil, an animal feed, in the pharmaceutical industries and also for biodiesel production [10,11]. It has an estimated availability of 1.5 million tons per year in India alone [12]. Its wide availability and similarity in fatty acid compositions and TG structure as CB prompted other applications of Sal fat. Since the early 1980s, Sal fat has been explored as a source of oil for CBEs [13]. However, its metabolic and physiological responses have been rarely investigated. Cocoa butter has a fatty acid profile of approximately 60% saturated fat, 35% monounsaturated fat and 1% polyunsaturated fat [14]. Similar to CB, Sal fat is high in saturated fat and is responsible for the structure integrity, mouth feel, texture and softness in chocolate confectioneries [6]. Promising results have been obtained, whereby Sal fat is able to substitute CB in any amount without altering the taste and texture of foods [12,15].

Although chocolates and cocoa-based products are praised for their ‘health benefits’, due to their polyphenolic content, the lipid content of chocolates has been a centre of attention, due to the putative link between dietary fat and health outcomes [16,17]. Due to its lower satiating effects compared to carbohydrates and protein, saturated fats have also shown to increase the risk of cardiovascular diseases and induce insulin resistance [18,19,20]. Despite the negative health implications of dietary fats on cardiovascular health, they play a vital role in the food system by being responsible for the sensorial properties of the food [21,22]. Hence, the development and application of fats and oil substitutes in food products remain a contentious challenge in modern society.

Two main scientific questions are addressed in this research: (1) Whether the substitution of CB with Sal fat altered the glycaemic, insulinaemic and lipidaemic responses in human subjects. (2) Whether the conversion of these oils into oleogel attenuated the glycaemic, insulinaemic and lipidaemic response. In recent years, a method used to convert liquid oils into solid form (oleogel) has gained traction and has been used to investigate the effects of postprandial glucose, insulin and TG responses in foods. A previous study demonstrated that transforming liquid oil into oleogel using ethylcellulose at approximately 150 °C reduced postprandial glucose and TG levels [23]. Incorporating oleogel into products is also an effective way to slow or prevent oil migration from cream fillings, which in turn prolongs shelf-life [14]. This significant finding and the application of lipid modification into oleogels could potentially be a solution to reduce the absorption of both saturated and trans fatty acids, recognizing how both these components increase the risk of cardiovascular diseases [24,25].

This is the first study to investigate the effects of Sal fat as a substitute for CB and its transformation into oleogels and the role that each lipid system has in effecting glycaemia, lipidaemia and insulinaemia.

## 2. Materials and Methods

This study was carried out at the Clinical Nutrition Research Centre (CNRC) within the Singapore Institute of Clinical Sciences (SICS), Agency of Science Technology and Research (A*STAR), Singapore. Ethical approval was received from the Domain Specific Review Board of the National Healthcare Group in Singapore (NHG DSRB Reference No. 2018/01193). Recruited participants gave written informed consent before the commencement of the study. All procedures and trial protocols were followed according to good clinical practice (GCP) guidelines and with the ethical standards in concordance to the Declaration of Helsinki, 1983. This study (registration no. NCT04115592) was registered under clinicaltrials.gov.

### 2.1. Study Population

Twenty healthy Chinese male adults were recruited from the general public using a variety of methods which included online advertisements, flyers and personal communication. Females were excluded from the study to eliminate potential variations in the results due to hormonal changes during females’ menstrual cycle.

Participants went through a screening of anthropometry (height, weight, waist/hip circumference), blood pressure and fasting blood glucose measurements. The inclusion criteria were healthy Asian Chinese males aged between 21 and 40 years, non-smoker, body mass index (BMI) 18.5–25.0 kg/m^2^, body weight of ≥45 kg and normal blood pressure of ≤140/90. Exclusion criteria were metabolic diseases (such as diabetes, hypertension, etc.), known glucose-6-phosphate dehydrogenase deficiency, and fasting blood glucose of >5.6 mmol/L in accordance with the guidelines of a healthy individual defined by the American Diabetes Association [26]. In addition, individuals with medical conditions and/or on prescription medications known to affect glycaemia (glucocorticoids, thyroid hormones, thiazide diuretics), allergies/intolerance to any of the test foods, competitive athletes and those with an intentionally restricting diet were excluded from the study, as these were shown to affect glucose and lipid metabolism [27].

### 2.2. Study Design and Study Protocol

The study was a randomised, controlled crossover design. Participants were required to attend four test sessions with a washout period of at least one week between test sessions. The four sessions included a chocolate brownie made from two different oil types (either a liquid or solid form). All screening and tests sessions were conducted at the CNRC.

Following an overnight fast, participants who expressed interests and met all inclusion criteria attended a screening session, where informed consent and anthropometric measurements were obtained. This study was a single blind design, as the blinding of the different states of oils was not possible. During screening, height was measured using a stadiometer (Seca Limited, Birmingham, West Midlands, Middlesex, UK), body weight and body composition was measured using the Bioelectrical Impedance Analysis (BIA) device (Tanita BC-418, Tokyo, Japan) and blood pressure was measured using the Omron blood pressure monitor (Model HEM-907). Fingerpick blood glucose was measured using the HemoCue 201+ Glucose RT analyser (HemoCue Ltd., Dronfield, UK), where the first two drops of expressed blood were discarded, and the next drop was collected directly into the microcuvette. The HemoCue has intra- and inter-assay coefficient of variation (CVs) of approximately 1.2% and 1.3%, respectively, for capillary blood glucose analysis. Thus, it is a widely accepted and reliable method for blood glucose assessments by the FAO/WHO [28].

Prior to the test day, participants were advised not to perform any strenuous physical activity and/or consume alcohol for 24 h. On the test day, participants arrived at the CNRC in the morning after a 10-h overnight fast. Upon arrival, participants rested briefly before an indwelling catheter was inserted into the antecubital vein and kept patent with 3 mL non-heparinised saline throughout the test session. Fasting venous blood sample (3 mL) was collected immediately after the insertion of the catheter. Participants were then told to consume the test meal provided within 15 min in an upright sitting position. Liking scores of the test meal was measured using a 100 mm visual analogue scale (VAS) anchored at ‘dislike extremely’ (−50 mm), ‘either like or dislike’ (0 mm) and ‘like extremely’ (+ 50 mm). Three millilitres of venous blood samples were taken at 15, 30, 45, 60, 90, 120, 150, 180, 210, 240, 270, 300, 330 and 360 min after the consumption of the test meal to determine plasma glucose, insulin and TG concentrations. The six-hour study period was selected, as this latency was required to observe maximal postprandial plasma TG concentration [14,23]. The participants were encouraged to minimise physical activity during the test period. The catheter was removed after 6 h and participants were free to leave the laboratory. The exact procedure was repeated for all four test sessions.

### 2.3. Test Meal

A standardised dinner was provided the day before the test session consisting of a Ready Meal (Butter chicken with cumin rice, Chef-in-Box, Singapore) and a packet drink (Milo Chocolate Malt, Nestlé, Switzerland).

Four different single test meals in a random order, consisted of: 

(1) Chocolate confectionery made from Cocoa Butter (CB)

(2) Chocolate confectionery made from Cocoa Butter oleogel (CBOG)

(3) Chocolate confectionary made from SAL seed oil (SL)

(4) Chocolate confectionery made from SAL seed oleogel (SLOG)

The oleogel was prepared by dissolving 11% *w/w* ethyl cellulose (viscosity 45 cP) to liquid oil at 140–150 °C (25 g liquid oil + 3.1 g ethyl cellulose). The mixture was left to cool to room temperature after ethyl cellulose was completely dissolved to form oleogel. The details of the oleogel preparation has been previously described [29]. The chocolate confectionery used in this study was brownies made from plain flour (Prima, Singapore), caster sugar (SIS, Singapore), cocoa powder (Hershey’s, Delhi, PA, USA), water, baking powder (Bake King, Singapore), vanilla essence (Bake King, Singapore), soy lecithin (Redman, Singapore) and baked at 160 °C for 45 min. In the SL and CB groups, 25 g of liquid oil was used, and 28.1 g of oleogel was used in the SLOG and CBOG groups. The oleogel was cut into small pieces and was evenly incorporated into the brownie. The four treatments were isocaloric (approximately 595 kcal; 50 g total available carbohydrates, 25 g fats) and participants remain fasted for the remaining 6 h.

### 2.4. Blood Analysis

To measure glucose and TG responses to test meals, venous blood samples were collected at fix intervals in Vacutainers^®^ (Belton Dickinson Diagnostics, NJ, USA) with disodium EDTA and centrifuged at 1500·*g* for 10 min at 4 °C (Sorvall^TM^ ST 16 Centrifuge, Thermo Fisher Scientific, Waltham, MA, USA). Plasma was then aliquoted into Eppendorf tubes and stored at −80 °C until analysis. Plasma insulin was measured using the immunochemistry analyser COBAS e411 (Roche, HITACHI, Los Gatos, CA, USA) while plasma glucose and TG concentrations were measured using the immunochemistry analyser COBAS c311 (Roche, HITACHI, Los Gatos, CA, USA). For insulin, the intra- and inter-assay CVs were <5% and <6%, respectively. For glucose and TG, the intra- and inter-assay CVs were <1.5% and <2%, respectively. The manufacturers provided all CVs of assays. Postprandial blood glucose concentration changes were determined by computing the difference between the fasting and the blood glucose concentration at a particular time point. The glycaemic response was obtained by using the changes in blood glucose concentrations to calculate the incremental area under the curve (iAUC) using a trapezoidal rule, ignoring the area beneath the baseline [30,31]. Changes from the baseline and iAUC were also calculated for insulin and TG responses.

### 2.5. Statistical Analyses

All statistical analyses were performed using the Statistical Package for Social Sciences software (IBM SPSS version 23.0; IBM Corp, Armonk, NY, USA) and statistical significance was set at alpha (α) 0.05, two-tailed. Data and figures were processed in a Microsoft Excel spreadsheet (Microsoft Corporation, Redmond, WA, USA). Values are presented as mean ± standard deviation unless otherwise stated. Prior to statistical analysis, the normality of the data was confirmed using the Shapiro–Wilk test. No data transformation was performed before the non-parametric analysis. Within and between treatment comparisons of means between test days for all variables were performed using the general linear model for repeated measures ANOVA with Bonferroni corrections. Tukey’s post-hoc analyses were conducted to compare differences between treatments at all-time points and were reported accordingly.

The sample size of this study was guided by our previous studies, where a statistically significant difference in blood TG and glycaemia was detected in 16 participants [23,32]. This was supported by the recommendations by FAO/WHO, where a minimum of 10 participants are required during the analysis of glycaemic, insulinaemic and lipidaemic responses in humans to account for inter-individual variations. An attrition rate of 15–20% was accounted for, thus bringing the total sample size to 20.

## 3. Results

### 3.1. Baseline Characteristics

Nineteen healthy male participants fulfilled the study inclusion criteria and were enrolled in the study. However, two participants withdrew from the study, due to personal reasons. Hence, no data was available for the subsequent analysis of those participants. Complete data was obtained for the remaining seventeen participants who completed all four treatments. The baseline anthropometric and biochemistry data is provided in Table 1. The anthropometric data were within normal ranges for all participants. The fasting glucose 5.57 mmol/L (*p* = 0.608), insulin 9.66 µU/mL (*p* = 0.454) and TG 0.798 mmol/L (*p* = 0.538) concentrations were not significantly different on the first test day for all treatments.

### 3.2. Metabolic Responses

**Figure 1:**
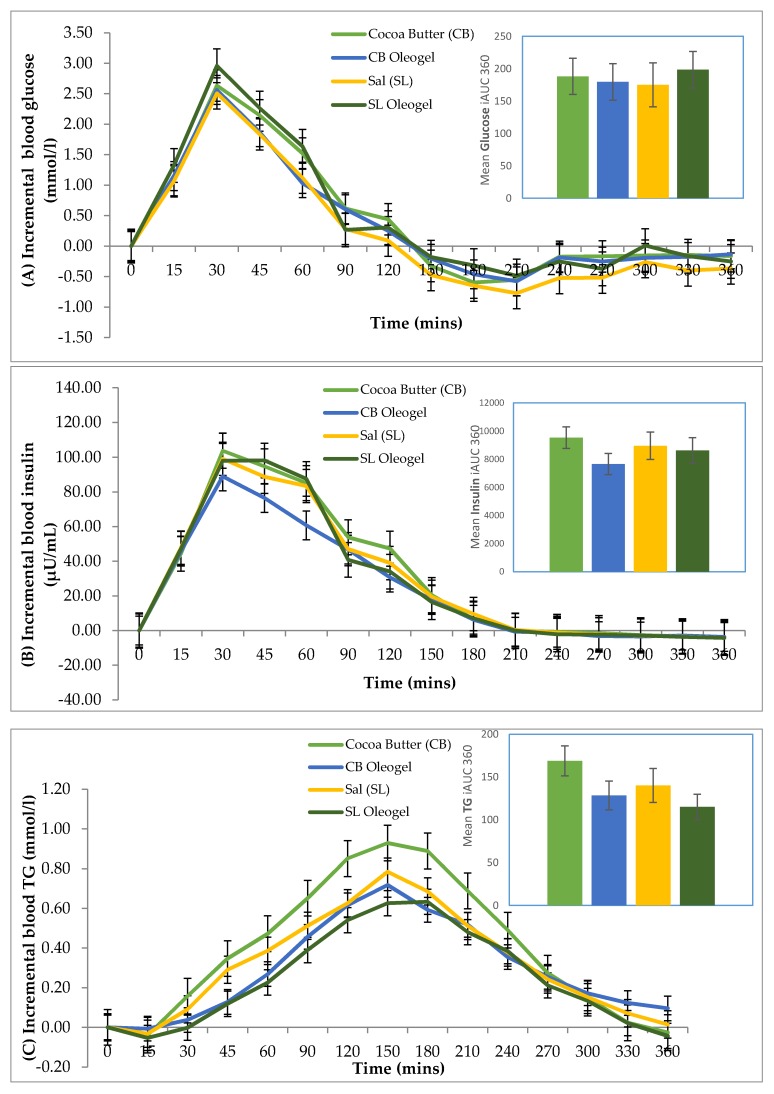
Postprandial incremental curves and area under the curve (iAUC) in glucose (**A**), insulin (**B**) and triglycerides (TG) (**C**) following the consumption of a chocolate brownie infused with cocoa butter (CB), Sal oil (SL), cocoa butter oleogel (CBOG) and Sal oleogel (SLOG). The bar plots displayed on the right of the graphs show the mean with error bars (SEM); *n* = 17. The mean iAUC360 relates to the area under the curve for the entire 360 min of measurement.

The postprandial changes in glucose (A), insulin (B), and TG (C) and the mean incremental area under the curve (iAUC) are graphically presented in Figure 1. Overall, there were no significant differences in the iAUC for blood glucose concentration (*p* = 0.995), plasma insulin (*p* = 0.760) and TG (*p* = 0.129) regardless of the oil type used. However, when comparing the iAUC of plasma insulin and TG between the different forms, it was found to be significantly different (*p* = 0.014 and *p* = 0.024, respectively), whereby the mean iAUC of oil in its gel form is lower than its liquid form. The mean iAUC of glucose concentration between the different forms of oil showed no significant effect (*p* = 0.669).

### 3.3. Liking Scores of Test Meal

The liking scores are as follows: CB = 66.68, CBOG = 60.82, SL = 65.62, SLOG = 67.06. They did not differ significantly when the scores were compared between both oils (CB and SL, *p* = 0.329) and when they were compared between the different forms (liquid and solid, *p* = 0.405).

## 4. Discussion

Our previous study demonstrated that coconut oil in oleogel form attenuated its effect on postprandial glucose and TG [23]. Hence, this study aimed to investigate whether the use of different oils as oleogel, incorporated into a chocolate brownie will also yield the same metabolic effects. The second aim of the study was to compare two fats of similar chemical structure, namely cocoa butter and Sal fat and their effects on postprandial glycaemia, insulinaemia and lipidaemia.

Similar to a previous study by Tan et al. (2017) [23], our results showed that there was a significantly lower increase in plasma insulin and TG of the brownie incorporated with CBOG and SLOG compared to the brownie with CB and SL. It is interesting to note that similar metabolic effects were obtained despite CB and SL containing higher amounts of palmitic and stearic acid, as compared to coconut oil, which contained mostly lauric and myristic acid (Table 2). The results from this study indicate that, independent of the degree of saturation or chain length, the conversion of oils into oleogels significantly attenuates postprandial insulin and TG levels.

Similarly, in another study, the degree of saturation of the oil, particularly palm oil and rice bran oil, showed no significant difference (*p* = 0.998) on postprandial plasma glycaemia and TG [37]. Hence, the results from these studies further support the notion that the physical form of the dietary fat (i.e., oil or as oleogel) plays a more significant role in its metabolic responses than the type or degree of lipid saturation.

Independent of the degree of saturation, it appears that when oils of varying degrees of saturation are transformed into an oleogel, the physio-chemical binding of the oils as an oleogel appears to ‘chelate’ the oil. Since the oleogel formed has ‘chelated’ the oils, the metabolic effect of the lipids influencing hyperlipidaemia appears to be blunted. Hence, ‘tropical oils’ that have been putatively blamed for the increase in hyperlipidaemia worldwide, may have this effect significantly reduced by the transformation of oils into oleogels.

Ethylcellulose, a semi crystalline derivative of cellulose used to encapsulate various pharmaceuticals, have been shown to also have a positive binding capacity to CB and SL despite having a higher degree of saturation (61.5% and 50.9% respectively), compared to palm oil and rice bran oil (49% and 25% respectively) [5,33,35,36]. As CB and SL are high in saturation, the oleogel formed is solid at room temperature. Therefore, heat treatment to the oleogel is necessary, prior to incorporating it into the brownie batter. It was observed that the physio-chemical binding structure of ethylcellulose to the oil was not disrupted even when heated. It was capable of reducing postprandial insulinemic and lipidemic responses. Therefore, the new ‘application of ethylcellulose’ to transform oils into oleogels makes it a highly valued food ingredient.

Results from our study also showed that there is no difference in metabolic effects between CB and SL, and between CBOG and SLOG. In addition, there is no difference in the liking scores of brownies amongst participants in all four treatments. This showed that Sal fat may be an acceptable substitute for cocoa fat in the production of chocolate confectioneries and showed no difference in their metabolic response. Therefore, these oils can be ‘interchanged’ with no public health concerns.

Chocolate confectioneries, including bakery products, are popular snacks consumed globally. They account for up to 43% of all cocoa consumed in 2017 [3,38]. The importance and the limitations of CB in the manufacturing of chocolates is widely recognized, as chocolate is a popular confectionery ingredient used worldwide, with increasing production growth in the last 10 years [39]. Research has shown that progressive climate changes will affect the production of cocoa in the time to come, in turn impacting majorly on food supplies, ecosystems, the livelihoods of farmers and environmental services [40,41]. Therefore, this is a noteworthy finding for food manufacturers and even healthcare professionals in the management of metabolic diseases arising from the consumption of these commonly consumed snacks.

## 5. Conclusions

We have demonstrated for the first time that Sal fat has no metabolic difference to CB. We have also shown that the use of different types of oil transformed into oleogels is effective in reducing postprandial insulinaemia and lipidaemia. Ethylcellulose is a viable ingredient to use in the manufacturing of oleogels that may be incorporated into various food products. Since it is heat stable and also able to be encompassed in a wide range of foods, it is likely that oleogels will become an increasingly popular ‘food ingredient’ in various food formulations. However, given that the sample size for this study is relatively small and that our work has been done only in a single meal, i.e., a chocolate brownie, the results from this study need to be interpreted with caution. More work needs to be performed in other food applications to further prove the heat stability of oleogel and its effects on postprandial metabolic responses. We also wish to further extend our activities to developing other formulations to manufacture oleogel using food ingredients other than ethylcellulose.

## Figures and Tables

**Table 1:** Baseline measurements of study participants (*n* = 17).

Characteristic	Mean ± SD
Age (years)	24.73 ± 2.63
Height (m)	173.81 ± 7.24
Weight (kg)	65.85 ± 8.06
BMI (kg/m^2^)	21.73 ± 1.65
Waist circumference (cm)	78.58 ± 9.48
Hip circumference (cm)	91.03 ± 20.21
Tricep skinfold (mm)	17.85 ± 7.83
Bicep skinfold (mm)	15.40 ± 10.25
Body fat (%)	16.52 ± 5.51
Systolic blood pressure (mmHg)	119.88 ± 6.90
Diastolic blood pressure (mmHg)	74.94 ± 8.13

**Table 2:** Fatty acid profiles of various types of oil (%).

Type of Oil	Saturated				Monounsaturated	Polyunsaturated
	Lauric Acid (C12:0)	Myristic Acid (C14:0)	Palmitic Acid (C16:0)	Stearic Acid (C18:0)	Oleic Acid (C18:1)	Linoleic Acid (C18:2)
Cocoa Butter [5]			25.1	36.4	34.1	2.8
Sal oil [33]			6.3	44.6	41.6	1.7
Coconut oil [34]	46.6	18.6	9.5	2.7	7.0	1.9
Palm oil [35]			45.0	4.0	40.0	10.0
Rice bran oil [36]			22.0	3.0	38.0	35.0
